# No country or continent is on its own in the ongoing COVID-19 pandemic

**DOI:** 10.2807/1560-7917.ES.2021.26.17.2100430

**Published:** 2021-04-29

**Authors:** Kari Johansen, Hanna Nohynek

**Affiliations:** 1 Independent public health expert, Stockholm, Sweden; 2 Finnish Institute for Health and Welfare (THL), Helsinki, Finland

**Keywords:** COVID-19 vaccines, vaccination, equity, evidence-based decision-making

Now 15 months into the coronavirus disease (COVID-19) pandemic, morbidity and mortality have been considerable and have affected people worldwide, irrespective of country of origin, socioeconomic status or genetic background. Strict lockdowns have lowered disease incidence and eased the burden on healthcare systems. Still, first data from early adapters of large-scale vaccination campaigns suggest that COVID-19 vaccines are the most effective measure against the disease and should allow for a gradual re-opening of society. However, a number of issues must be resolved before life similar to before the pandemic can resume. This year’s European Immunization Week, from 26 April to 2 May, is an opportunity to celebrate the achievements of the COVID-19 pandemic response, as well as discuss the remaining challenges in Europe and globally, such as limited vaccine availability, lack of equitable vaccine distribution and the implications of the emerging severe acute respiratory syndrome coronavirus 2 (SARS-CoV-2) variants of concern (VOCs).

Following the identification of SARS-CoV-2 in early January 2020 [1], vaccine candidates began to be developed at an unprecedented pace. A year later, over 10 candidates have received emergency use approvals or conditional marketing authorisations by either national regulatory agencies, the European Medicines Agency (EMA) or the World Health Organization (WHO) prequalification system. On 27 April 2021, WHO listed 276 vaccine candidates in its global COVID-19 candidate vaccine landscape and tracker, of which 92 are being tested in clinical phase 1, 2 or 3 trials [2]. Among the 10 different vaccine production technology platforms listed, the most common are protein subunit, non-replicating viral vectors, inactivated whole virion and mRNA vaccines, with the protein subunit vaccines in need of adjuvants. The recommended priming schedules vary between administration of 1 to 3 doses, delivered 2 to 12 weeks apart, with the most common schedule being 2 doses administered 3 to 4 weeks apart. The route of administration varies from intramuscular to intradermal, subcutaneous, oral or intranasal.

The urgency of the pandemic led to a paradigm shift in vaccine clinical trials. The different trial phases were run in parallel rather than in sequence, and national regulatory authorities and the EMA agreed to intensive advising and rolling review, to speed up the licensure process. An a priori threshold for approval of 50% efficacy, with a lower bound of 95% confidence interval of 30%, was accepted [3]. For safety, a minimum period of 2 months of follow-up was agreed, which is justified, as most known adverse events are recognised within 6 weeks from vaccination. By April 2021, four COVID-19 vaccines received conditional marketing authorisation in the European Union (EU) [4] and a rolling regulatory review has been initiated for four additional vaccine candidates, adding further production technologies to the portfolio.

Several of the globally authorised vaccines, especially the mRNA and adenovirus-vectored ones, have exceeded expectations and protect remarkably well against death and severe disease. After only one dose, significant protection against hospitalisation was observed 4 weeks post immunisation, for both vaccine types [5,6]. In addition, these vaccines protect against infection and reduce viral load, thereby likely also reducing transmission and contributing to indirect protection in the community [7].

Vaccine candidates have been developed and associated clinical trials have been conducted with considerable public and private donations through national governments, institutional funders and others, in collaboration with smaller- and larger-scale academic, governmental and private vaccine developers. The rapid development benefited from many years of basic research establishing vaccine production technology platforms for similar pathogens, in particular severe acute respiratory syndrome coronavirus 1 (SARS-CoV-1) and Middle East respiratory syndrome coronaviruses (MERS-CoV). Through the joint EU procurement mechanism, all four conditionally authorised vaccines have been delivered to all EU countries, not necessarily pro-rata but in different proportions and in line with country requests, which have differed, as evidenced in the European Centre for Disease Prevention and Control’s (ECDC) vaccine tracker on the COVID-19 vaccines distributed in the EU/European Economic Area (EEA) countries [8].

In many countries, recommendations on vaccine use in the national programmes are based on grading the available scientific evidence in a systematic and transparent manner (Table 1). Depending on the funding basis of the programme, cost-effectiveness analyses may also be applied to support decision-making. However, for prevention of COVID-19, cost has—for most EU countries—been a secondary issue at the national level due to the EU joint procurement mechanism, though this is different at the global level, both in the bilateral agreements and through the COVID-19 Vaccines Global Access (COVAX) mechanism [9]. Globally, the WHO Strategic Advisory Group of Experts (SAGE) has tried to guide countries that are introducing COVID-19 vaccines while facing a supply shortage. The ´WHO SAGE values framework for the allocation and prioritisation of COVID-19 vaccination´ emphasises the six common values that countries should respect while planning the deployment of COVID-19 vaccines: human well-being, equal respect, global and national equity, reciprocity and legitimacy [10]. Further, the ´WHO SAGE Roadmap For Prioritizing Uses Of COVID-19 Vaccines In The Context Of Limited Supply - An approach to inform planning and subsequent recommendations based upon epidemiologic setting and vaccine supply scenarios´ draws three basic scenarios on how to use the value framework mentioned above in different epidemiological settings, with low-, middle-, and high-circulation of SARS-CoV-2 [11]. In all of these scenarios, frontline healthcare workers and older adults are the most vulnerable population groups and should be vaccinated first.

**Table 1:** Evidence and factors to be considered in national vaccination decision-making

Evidence to recommendation domain	Question
Public health problem	Is the problem of public health importance?
Benefits and harms	How substantial are the desirable anticipated effects? How substantial are the undesirable anticipated effects? Do the desirable effects outweigh the undesirable effects?
Values	Does the target population feel the desirable effects are large relative to the undesirable effects? Is there important variability in how patients value the outcomes?
Acceptability	Is the intervention acceptable to key stakeholders?
Feasibility	Is the intervention feasible to implement?
Resource use	Is the intervention a reasonable and efficient allocation of resources?
Equity	What would be the impact of the intervention on health equity?

Adapted from the Standard Operating Procedure of the German Standing Committee on Vaccinations (STIKO) for the systematic development of vaccination recommendations [31].

National Immunization Technical Advisory Groups (NITAGs) or equivalent committees have provided advice to their governments using these WHO SAGE frameworks and their own decision-making processes on how to best use these new vaccines. In Europe, the EU/EEA NITAG Collaboration network [12], coordinated by ECDC, has provided a platform for useful exchange among those in charge of expert advice for national vaccination programmes. However, as COVID-19 epidemiology and disease burden have varied across countries since the beginning of the pandemic, there have been differences between countries in interpreting benefit–risk ratios and deciding on vaccination prioritisation order. This was observed in national decisions relating to the early use of Vaxzevria (formerly COVID-19 Vaccine AstraZeneca, AstraZeneca, Cambridge, University of Oxford, Oxford, United Kingdom (UK)), in the absence of robust trial data among older adults (> 65 years old), and again when some countries made changes to their vaccination programmes after the vaccine safety signal noting reports of rare thrombotic thrombocytopenic syndrome among those administered the same vaccine emerged [13-16]. Both of these examples have contributed to the current differences between country-specific recommendations for this vaccine in the EU.

The European Commission has set the target of 70% coverage of a first dose of COVID-19 vaccines in all countries by July 2021. As at the end of April 2021, the cumulative uptake of 26.5% for at least one vaccine dose in the EU [8] is lower than in a few other high-income countries like Israel, the UK and the United States (US) [17]. However, from a global perspective—with an average of 7.4% for the first dose, as at 26 April 2021 [17]—most EU countries are doing well. Vaccination programmes have also started in all non-EU countries in the WHO European Region, while introduction of COVID-19 vaccines is still pending in 14 other countries worldwide [17]. Whether vaccine coverage can reach a high enough threshold to provide community immunity in the EU, remains to be seen.

Newly emerging SARS-CoV-2 variants that could be capable of escaping vaccine protection may present a challenge to COVID-19 control and the future scenario may be similar to what we observe with vaccines against influenza, where we seldom expect community immunity. Based on its experience of routinely updating seasonal influenza vaccine strains, WHO is currently developing a framework for assessing new SARS-CoV-2 variants that will contain a process for variant selection to be included in future multivalent COVID-19 vaccines, if found to be safe and effective in the ongoing clinical trials [18-20]. This global framework is not ready yet, but regulatory agencies such as the EMA and the US Food and Drug Administration have developed guidelines for vaccine developers on how to test updated COVID-19 vaccines [21,22]. Further, many EU countries have made breakthrough infections following vaccination notifiable, and it is important that SARS-CoV-2 viruses that are able to cause infection despite prior vaccination undergo whole genome sequencing. Safety and effectiveness studies of COVID-19 vaccine in use are also being set up using different study designs under the new EMA-ECDC collaboration framework that will address variant-specific performance [23].

Even once 70% COVID-19 vaccine coverage among adults is reached, there are additional aspects that need careful consideration: waning immunity, the role of children and adolescents in disease transmission, availability of vaccines for children and guidance on how to best use vaccines in pregnant women, as well as immunocompromised individuals. Whether booster doses will be needed for all or some of the vaccine candidates is currently uncertain. Clinical trials have been initiated with candidates containing one or several of the newly emerged variants to possibly be used in yearly boosters or more often, if needed. Several manufacturers are far advanced in developing childhood COVID-19 vaccines, and the first extended vaccine indication for one or several vaccines may come as early as the second quarter of 2021 for children aged 12–15 years. However—above all—community immunity through a combination of vaccine-induced immunity and natural immunity following infection will not provide full protection given the disparities in vaccine coverage from region to region and serious global vaccine shortages; it will take several years to reach a minimum of 70% coverage worldwide.

First laboratory studies and limited effectiveness studies suggest that the EU-authorised vaccines are protective against the B.1.1.7 variant first identified in Kent, UK, but are less protective against the B.1.351 and P.1. variants, first identified in the Cape region of South Africa and Manaus, Brazil, respectively [24,25]. There are no results available yet for the most recently identified new variants from India, B.1.617 and its subclades, which have more genetic changes compared with any other globally identified variant. The risk of breakthrough infection with the newly circulating SARS-CoV-2 VOCs in previously infected or vaccinated individuals is why all countries, including those with high vaccination coverage, may still maintain the non-pharmaceutical interventions used up to now, in order to offer individual- and population-level control against these variants. Guidance on how to safely ease control measures in exit strategies is already available, but when such measures can be safely lifted remains uncertain in the rapidly changing epidemiological landscape [26].

Within this context, one important population group to be monitored is immunocompromised people. They are expected to respond worse to vaccination, which may give rise to new SARS-CoV-2 variants if they are carrying virus for longer time periods. They may thus need effective treatments, e.g. in the form of antivirals, and effective antivirals will also be necessary to treat individuals hospitalised with severe COVID-19 disease.

As mentioned above, there are many remaining challenges in Europe and globally. Table 2 gives an overview of some important identified challenges and lists activities that are a shared responsibility between the national, regional and global levels using standardised methodology to allow for comparability of data collected.

**Table 2:** Future challenges identified and necessary monitoring and evaluation activities to guide the evidence-based short- and long-term vaccine and vaccination response to the COVID-19 pandemic in the European Union/European Economic Area and globally

Domain	Identified challenges	Necessary monitoring and evaluation activities
Epidemiology	Routinely monitoring infected cases, hospitalised cases, cases in intensive care and mortality	Ensure timely reporting
	Reports on age and risk groups for respective category above	Ensure timely reporting
	Reports on co-infections (e.g. SARS-CoV-2 + influenza, RSV or measles upon return)	Ensure timely reporting
	Identify changes in epidemiology such as new clusters or increased severity in a timely manner	Ensure timely investigations and reporting on epidemiological changes at the EU/EEA level and globally that may impact vaccine composition or vaccination recommendations
Circulating SARS-CoV-2 viruses	Availability of diagnostics	Ensure diagnostics offered generously and results reported in a timely manner Ensure diagnostics of possible co-infections
	Identification and monitoring incidence of SARS-CoV-2 variants of interest and concern	Whole genome sequencing of representative samples and timely reporting
	Assessment of transmissibility, severity and vaccine escape	Ensure a functioning EU/EEA-level and support to a global framework for rapid assessments of circulating variants of interest and concern in a timely manner
	Assessment of need for updated vaccines	Support to a global framework for rapid assessment of new variants of concern and timely recommendations for updates of COVID-19 vaccines
Vaccine availability	Timely vaccine development	Support to continued vaccine development, clinical trials and if needed updated vaccines in response to new emerging SARS-CoV-2 variants of concern
	Vaccine production capacity to fulfil public health recommendations	Map and ensure production and filling capacity in a sufficient number of EU/EEA sites to serve the EU/EEA population Ensure access to raw materials for vaccine production
	National and EU equity	Map and ensure vaccine equity within and between EU/EEA countries
	Global equity	Map and ensure vaccine equity within and between countries globally
Vaccine performance	Vaccine coverage	Ensure routine product-specific documentation of COVID-19 vaccines administered to individual citizens in immunisation registries available over time to support decisions on administration of booster doses, if needed
		Timely reporting of vaccination coverage by age and risk groups at national and EU levels
	Vaccine safety	Ensure routine safety monitoring and timely reporting and publishing in a transparent manner at EU/EEA level
		Ensure vaccine safety signal detection system and validation at the EU/EEA level
		Develop case definition if unknown adverse event
		Ensure background incidence rates available for all adverse events of special interest in a representative number of EU/EEA countries for observed versus expected analysis and development of regulatory assessments of benefit–risk ratio
		Ensure conduct of product-specific pharmacoepidemiologic studies to be reported to public health and regulators in a timely manner and published in a transparent manner
		Explore if product-specific, real-time scientific studies can be performed
		Ensure vaccine safety communication channels are in place should a vaccine safety signal occur
	Vaccine effectiveness	Ensure conduct of product-specific vaccine effectiveness studies addressing outcomes such as any COVID-19 infection, hospitalisation due to COVID-19, death due to COVID-19, long COVID-19, transmission and viral shedding by SARS-CoV-2 variants to be reported to public health and regulators in a timely manner and published in a transparent manner
		Ensure conduct of product-specific vaccine effectiveness studies in special groups (comorbidities, children, pregnant women, immunocompromised, etc.)
		Ensure conduct of product-specific vaccine effectiveness studies addressing different time intervals between dose 1 and 2
		Ensure conduct of product-specific vaccine effectiveness studies addressing mix and match of authorised COVID-19 vaccines
		Explore if product-specific real-time effectiveness studies can be performed
	Vaccine escape	Ensure seroepidemiological studies to monitor for waning immunity in immunised populations
		Ensure immunological studies, including neutralising antibody assays for new SARS-CoV-2 variants of concern
	Vaccine breakthrough infections	Ensure routine monitoring of breakthrough infections through notifiable systems and ensure timely reporting

COVID-19: coronavirus disease; EU/EEA: European Union/European Economic Area; RSV: respiratory syncytial virus; SARS-CoV-2: severe acute respiratory syndrome coronavirus 2.

While the world has been focused on COVID-19, the incidence of other respiratory pathogens such as seasonal influenza and respiratory syncytial virus (RSV) has diminished in many countries, most likely thanks to non-pharmaceutical control measures. Once life returns to normal—or closer to normal—these pathogens will return as well. While healthcare workers and public health efforts have been focused on COVID-19–related activities, other sectors have suffered. Less attention to other vaccines in the national programmes and increased disparities as a result of the pandemic may lead to future outbreaks of other diseases. In this issue of *Eurosurveillance*, Rohleder et al. report that the risk of measles incidence in areas with the lowest deprivation quintile was 1.58 times higher (95% credible interval (CrI): 1.32–2.00) than in those with highest deprivation in Germany [27]. In the post-COVID-19 pandemic world, we need to be vigilant with these diseases. On the other hand, the pandemic has forced us to build and improve our adult vaccination infrastructure, as pointed out by Williams et al. in their global review, wherein they found that the Americas and Europe had the highest proportions of adult immunisation programmes, most commonly for hepatitis B vaccine and influenza vaccines (> 47% and > 91% of countries, respectively) [28]; however, now is the time to strengthen adult immunisation programmes and programmes targeted at healthcare workers, overall. Assessing protective immunity in healthcare workers for diseases with known correlates of protection is one possible strategy and opportunity, as shown in the article by von Linstow et al., also in this issue [29].

## Conclusions

During the COVID-19 pandemic, important lessons for the future have been learnt on rapid vaccine development, authorisation, procurement, distribution and administration in large vaccination campaigns. Careful assessments of which technologies can provide safe and effective vaccines for all age and risk groups in the timeliest manner is now key moving forward.

Clinical trial networks conducting rapid trials taking into account the VOCs; genetic differences between global populations; and different age and risk groups, including underlying conditions such as obesity, being immunocompromised and other factors potentially influencing vaccine response, will likely also play an important role in future.

The possibility to administer COVID-19 vaccines to children and adolescents, who constitute a large part of the population worldwide, in some countries almost half of the population, should allow for a notable reduction in global transmission. Only after children and adolescents, as well as pregnant and lactating women, can safely be offered COVID-19 vaccines will it be likely that indirect protection is provided to groups that are unable to receive the vaccines themselves, such as those who are immunocompromised [30].

It should be noted that long-term safety and effectiveness are still unknown for the current and soon-to-be authorised vaccines in the EU and will guide the choice of vaccines to be recommended long-term.

The lessons from COVID-19 vaccination should help us to further expand adult immunisation programmes in general—and, in particular, for healthcare workers—beyond hepatitis B and influenza to pneumococcal, pertussis, herpes zoster and other future vaccines already under development, such as those against RSV.

Exploring further how vaccines can be produced and distributed in an equitable manner is also necessary, since it is clear that no country or continent is on its own in ending the COVID-19 pandemic; international collaboration and global capacity building will be necessary to achieve this. Producing enough COVID-19 vaccines to equally serve every country in the world is an enormous challenge. Collaboration between vaccine developers, manufacturers, regulators, public health experts, governments and donors around the world will be key for continued vaccine development and global capacity building for vaccine production and deployment in all continents.

Finally, all of the decisions necessary for society to open up again should be evidence based at every step. To allow for such decision-making, well-designed surveillance systems and properly funded studies are necessary to critically appraise all implemented public health measures in the years to come. The tools to curb the COVID-19 pandemic are available to us, but we need to regularly assess them and use them in a scientifically sound manner.
